# Expert Opinion on the First-Line Management of Neovascular Age-Related Macular Degeneration and Diabetic Macular Edema in India: The Role of Faricimab

**DOI:** 10.7759/cureus.100779

**Published:** 2026-01-04

**Authors:** Chaitra Jayadev, Dinesh Talwar, Muna Bhende, Raja Narayanan, Vishali Gupta, Debdulal Chakraborty, Satyen Deka, George Manayath, Alay Banker

**Affiliations:** 1 Vitreo Retina, Narayana Nethralaya Eye Institute, Bengaluru, IND; 2 Vitreo Retina, Center For Sight, Delhi, IND; 3 Vitreo Retina, Sankara Nethralaya, Chennai, IND; 4 Vitreo Retina, Flaum Eye Institute, University of Rochester, New York, USA; 5 Vitreo Retina, Post Graduate Institute of Medical Education and Research, Chandigarh, IND; 6 Vitreo Retina, Disha Eye Hospital, Hooghly, IND; 7 Vitreo Retina, ASG Eye Hospital, Guwahati, IND; 8 Vitreo Retina, Aravind Eye Hospital, Coimbatore, IND; 9 Vitreo Retina, Banker Retina Center, Ahmedabad, IND

**Keywords:** angiopoietin-2, diabetic macular edema, faricimab, neovascular age-related macular degeneration, treat-and-extend regimen

## Abstract

This expert opinion article summarizes deliberations from a focused panel discussion involving nine retina specialists on the clinical utility of faricimab in managing neovascular age-related macular degeneration (nAMD) and diabetic macular edema (DME). The discussion explored evolving understanding of the multifactorial disease mechanisms, including pathways beyond vascular endothelial growth factor-A, and the rationale for dual inhibition strategies. Experts shared their clinical perspectives on faricimab use in diverse patient profiles, treatment sequencing, and individualized dosing approaches to achieve durable disease control. Practical considerations around early initiation, optimization of treatment intervals, and real-world applicability were also discussed. The insights highlight expert-derived strategies for integrating faricimab as a first-line treatment into clinical practice, emphasizing its role in achieving vascular stability and the improved durability of treatment response. Overall, the panel concluded that faricimab represents a clinically valuable first-line therapeutic option for nAMD and DME, enabling individualized, durable disease control in routine practice.

## Introduction and background

Neovascular age-related macular degeneration (nAMD) is an advanced form of AMD characterized by choroidal neovascularization beneath the macula, leading to rapid and often irreversible vision loss. Globally, AMD affects approximately 196 million individuals, with projections exceeding 243 million by 2030. In India, late-stage nAMD affects 0.11%-1.2% of the population [1-5]. Polypoidal choroidal vasculopathy (PCV), a common nAMD subtype in Asian populations, accounts for 22.3%-61.6% of cases and contributes substantially to disease burden [6,7]. Vision loss in nAMD typically results from subretinal hemorrhage, fibrosis, and scarring [8].

Diabetic macular edema (DME) is a major cause of vision loss among individuals with diabetes, arising from retinal vascular leakage and macular thickening [9]. In India, the prevalence of diabetes is 11.4%, and an estimated 1.54 million individuals have treatable DME [10-12]. Without timely treatment, DME leads to progressive central vision impairment and reduced quality of life.

Both nAMD and DME share pathophysiological mechanisms driven by increased vascular permeability. While vascular endothelial growth factor (VEGF) is central to disease progression, vascular stability is also regulated by the angiopoietin (Ang)-Tie2 signaling pathway. Elevated Ang-2 disrupts Tie2 signaling, amplifying VEGF-mediated leakage, inflammation, and neovascularization [9]. Diagnosis relies on multimodal retinal imaging, including fundus examination, optical coherence tomography, and fluorescein angiography [13].

In India, approved anti-VEGF therapies for nAMD and DME include ranibizumab, aflibercept, and brolucizumab [9]. Faricimab, a bispecific antibody targeting both VEGF-A and Ang-2, was approved in India in April 2023 and was launched in January 2024. It has received global approvals in 107 countries as of February 2025 [14-16]. Dual inhibition with faricimab improves vascular stability, reduces retinal fluid, and enables extended dosing intervals, supporting favorable anatomical and visual outcomes [17].

Management of retinal diseases in India is further challenged by delayed diagnosis, limited access to care, and poor long-term adherence. Therapies with greater durability and reduced injection frequency may help address these barriers by lowering treatment burden in real-world and resource-constrained settings [8,9,17,18].

## Review

Methodology

A moderated expert panel of nine retina specialists from India, with extensive experience in nAMD, DME, and PCV, convened to discuss dual VEGF-A/Ang-2 inhibition with faricimab. The panel reviewed disease burden, treatment challenges, mechanistic rationale, clinical trial evidence, and real-world outcomes in India. Discussions were structured into nAMD and DME sessions and focused on clinical and real-world implications, dosing strategies, and treatment durability. Insights from published studies, pivotal trials, and real-world evidence were presented, transcribed, and synthesized into thematic recommendations that reflect expert consensus, rather than a systematic review. The moderator facilitated a structured discussion, ensuring coverage of treatment challenges, molecular mechanisms, efficacy outcomes, dosing intervals, safety, and healthcare access considerations in India. Panel insights were recorded and analyzed to provide a comprehensive narrative of faricimab’s clinical positioning.

Role of VEGF-A and Ang-2 in the pathogenesis of retinal vascular diseases

VEGF-A promotes angiogenesis and vascular permeability, and Ang-2 disrupts vascular stability via the Tie2 receptor. Preclinical ischemic retinal and choroidal neovascularization models have demonstrated that coexpression of VEGF-A and Ang-2 accelerates lesion development, whereas suppression of Ang-2 stabilizes vessels and promotes regression. In diabetic retinal disease, elevated Ang-2 levels are associated with progression to proliferative disease. Murine models have demonstrated that simultaneous inhibition of VEGF-A and Ang-2 is more effective than anti-VEGF monotherapy, resulting in reduced vascular leakage, retinal edema, neurodegeneration, lesion size, macrophage infiltration, and apoptotic markers [7,19].

In India, the high prevalence of PCV and the growing diabetes burden underscore the need for therapies addressing multiple pathogenic pathways. Inhibition of both VEGF-A and Ang-2 improves vascular stability, reduces leakage and inflammation, and provides durable anatomical and functional outcomes, as demonstrated in both preclinical and clinical studies [7,20-24]. Post hoc and exploratory analyses further suggest that these benefits may be clinically meaningful in chronic or refractory disease [25]. Preclinical studies also suggest the potential for preventing astrocyte apoptosis, which could limit retinal neurodegeneration in DME [25].

The Phase 3 YOSEMITE and RHINE trials have demonstrated reduced fibrotic remodeling and long-term preservation of retinal architecture with faricimab [7]. Mechanistic studies have further demonstrated the suppression of nuclear factor kappa B (NF-κB) signaling and leukocyte infiltration, which contribute to reduced inflammation and vascular damage [7]. Meanwhile, imaging data indicate faster resolution of macular edema with faricimab than with single-pathway agents [26,27]. The key mechanistic pathways, associated biomarkers, and clinical effects are summarized in Table 1 [28].

**Table 1:** Role of VEGF and Ang-2 in retinal disease pathogenesis and clinical biomarker control with dual VEGF/Ang-2 targeting Ang-2: Angiopoietin-2; DME: Diabetic macular edema; ERM: Epiretinal membrane; HRF: Hyperreflective foci; IRF: Intraretinal fluid; nAMD: Neovascular age-related macular degeneration; OCT: Optical coherence tomography; PED: Pigment epithelial detachment; Q12W: Every 12 weeks; RVO: Retinal vein occlusion; VEGF: Vascular endothelial growth factor.

Pathway/biomarker elevated	Pathophysiologic feature	Clinical evidence/effect observed with faricimab
VEGF	Neovascularization	• Greater reduction of fluid on OCT (DME, nAMD) • Greater resolution of IRF (DME) • Greater resolution of PED (nAMD)
	Vascular leakage	• Greater reduction of macular leakage (DME, RVO) • Greater reduction of hard exudates (DME)
Ang-2	Inflammation	• Greater reduction of HRF (DME)
	Fibrosis	• Greater reduction of ERM development (DME)
Overall outcome of dual pathway inhibition (VEGF + Ang-2)	—	• Improved disease control vs. anti-VEGF monotherapy • Extended durability beyond Q12W dosing

Building on preclinical and biomarker-driven insights, the vascular stabilization and anti-inflammatory effects observed with faricimab have translated into significant improvements in real-world endpoints, including visual acuity, retinal thickness, and treatment durability. As detailed in the following section, these mechanistic insights underpin the therapeutic profile of faricimab in the real-world management of retinal disease.

Impact on visual acuity and durability

Indian patients often face multiple challenges with anti-VEGF therapies, including limited access to specialized care and difficulties in maintaining frequent follow-up visits. These barriers contribute to poor adherence and suboptimal outcomes. Faricimab's extended dosing intervals (up to 16 weeks) represent a significant advancement in this context, reducing both treatment burden and financial strain in resource-limited settings. Accordingly, treatment durability has emerged as a clinically meaningful endpoint in real-world decision-making.

Faricimab demonstrated significantly improved visual acuity and anatomical outcomes across pivotal trials, including YOSEMITE, RHINE, TENAYA, and LUCERNE. In DME, visual acuity gains and extended dosing intervals (up to 16 weeks) were sustained for a period of 2 years. Similarly, in nAMD, faricimab provided lasting improvement in visual acuity with longer treatment intervals.

Durability is supported by trial data, with nearly 80% of patients achieving ≥12-week treatment intervals by Week 112 in the TENAYA and LUCERNE trials, and the majority of patients in the YOSEMITE and RHINE trials maintaining every 12 weeks (Q12W) or every 16 weeks (Q16W) dosing up to Week 96 [26,29]. These durability outcomes were achieved without compromising visual acuity or anatomical control.

In addition to its dosing flexibility, faricimab has demonstrated superior anatomical outcomes compared to aflibercept. In the head-to-head phase of the TENAYA and LUCERNE trials, faricimab resulted in a greater reduction in central subfield thickness (CST) by Week 12, reflecting a faster and more effective resolution of retinal edema [30]. Furthermore, it was noted that during the 12‑week head‑to‑head induction phase, faricimab and aflibercept achieved virtually identical clearance of intraretinal fluid (IRF) (≈88% vs. ≈85% at Week 12). In addition, faricimab demonstrated significantly greater subretinal fluid (SRF) resolution and combined IRF + SRF absence by Week 12 (≈88% vs. 79% SRF‑free; ≈77% vs. 67% completely fluid‑free; both nominal p<0.001) [30].

By improving vascular stability and fluid control, faricimab provides consistent anatomical and functional benefits that translate into durable visual outcomes. In India, where healthcare resources are often limited, the ability to maintain disease control with extended dosing intervals is particularly relevant, as it reduces the frequency of visits while preserving efficacy. The safety profile of faricimab, including common ocular adverse reactions and potential risks observed across pivotal clinical trials, is summarized in Table 2.

**Table 2:** Common adverse reactions and risks associated with faricimab IOP: Intraocular pressure; VEGF: Vascular endothelial growth factor.

Category	Adverse reaction/risk	Clinical context/evidence
Ocular (common)	Conjunctival hemorrhage	Reported across TENAYA, LUCERNE, YOSEMITE, and RHINE trials; generally mild and self-limiting
	Increased IOP	Transient IOP elevations observed after injection, consistent with intravitreal therapies
	Vitreous floaters	Injection-related; comparable with other intravitreal anti-VEGF agents
	Eye pain/irritation	Mild-to-moderate; typically resolves without intervention
Ocular (less common but serious)	Intraocular inflammation	Low incidence; comparable with aflibercept in pivotal trials
	Retinal vasculitis/retinal artery occlusion	Rare; monitored closely after marketing; no excess risk observed vs. comparator arms
	Endophthalmitis	Very rare; associated with the intravitreal injection procedure rather than a drug-specific mechanism
Systemic	Arterial thromboembolic events	Low incidence; no significant imbalance compared with aflibercept in Phase 3 trials
Overall safety profile	—	Safety profile comparable to that of established anti-VEGF therapies, with no new safety signals identified in pivotal trials

Expert panel overview and discussion framework

A focused expert panel discussion was held in Guwahati, Assam, featuring nine retina specialists from India with extensive experience in managing macular diseases, including nAMD, PCV, and DME. The aim was to gain expert recommendations on the evolving role of faricimab as a first-line treatment for nAMD and DME. All panel members had significant experience with a wide range of intravitreal pharmacotherapies. The existing published literature and the panel's collective clinical experience steered the discussion. Key areas explored included current challenges in intravitreal therapy for nAMD and DME, the molecular mechanisms and clinical impact of existing anti-VEGF agents, anatomical and functional outcomes with conventional vs. newer agents, particularly faricimab, and the comparative value of clinical trial data vs. real-world evidence. The panel also evaluated the long-term efficacy and durability of newer therapies, outcomes in PCV (noting its high incidence in the Indian population), optimal treatment strategies for extended intervals with minimal interventions, safety considerations of newer agents, and specific barriers to effective treatment in the Indian context. These discussions formed the basis for the expert recommendations provided. The conversations were transcribed, analyzed, and synthesized into this comprehensive report.

This manuscript summarizes the outcomes of an expert panel discussion and does not include any human or animal research, patient data, identifiable clinical information, or interventional research. Therefore, institutional ethics committee approval was not applicable. All panel experts provided informed consent to publish their views, as shared in the discussion, to facilitate scientific dissemination.

Review of evidence with expert insights

This section synthesizes data from pivotal clinical trials and real-world studies that demonstrate faricimab's ability to address the limitations of current anti-VEGF therapies by providing durable efficacy, extended treatment intervals, and a favorable safety profile. The discussion is structured to explore the pathophysiological rationale, clinical outcomes, and expert consensus on faricimab's evolving role, particularly within healthcare settings like India.

Dual pathway inhibition in retinal disease pathophysiology

Pathological conditions, such as hypoxia, hyperglycemia, and oxidative stress, upregulate Ang-2, destabilizing endothelial integrity and promoting inflammation by disrupting the blood-retinal barrier [31-34]. VEGF exacerbates vascular leakage and neovascularization, and Ang-2 antagonizes the protective Tie2-Ang-1 signaling axis, further compromising vascular stability [18,35]. Standard anti-VEGF therapies address VEGF-driven pathology but do not target Ang-2-mediated destabilization, potentially limiting disease control. Faricimab addresses this gap and provides durable vascular stabilization. Its action reduces pericyte loss, limits vascular permeability, and mitigates inflammation [36,37]. Post hoc analyses of Phase 3 data have demonstrated superior biomarker resolution, including reductions in hyperreflective foci, epiretinal membrane risk, and persistent IRF, supporting improved anatomical and functional outcomes [16,28,36,38,39].

Addressing unmet needs in anti-VEGF therapy

In resource-limited settings, the ability to extend treatment intervals without compromising efficacy has significant implications for healthcare delivery and patient outcomes. Despite the effectiveness of anti-VEGF monotherapy, unmet needs persist. Monthly injections and clinic visits impose a high treatment burden, leading to poor adherence and suboptimal visual outcomes in real-world settings [33]. Studies have shown that over 50% of patients discontinue therapy within 24 months, with non-adherence rates ranging from 32% to 95% [40]. Factors such as older age, lack of caregivers, comorbid illness, and inadequate patient education contribute significantly to both non-adherence and non-persistence.

In this context, faricimab's extended dosing intervals offer a critical advantage. Experts recommend faricimab for its ability to provide extended dosing intervals of up to 16 weeks, thereby reducing the frequency of visits and improving patient adherence [26,29]. In the YOSEMITE and RHINE trials for DME, over 90% of patients treated with faricimab achieved CST <325 µm and maintained best-corrected visual acuity (BCVA) gains, with dosing intervals extended up to every 16 weeks [26,29]. These results were confirmed in the RHONE-X extension study, where the durability and safety of faricimab remained consistent [41]. Similarly, in the TENAYA and LUCERNE trials for nAMD, ≥74% of patients achieved Q12W or Q16W dosing intervals with comparable visual gains to aflibercept [29].

Real-world evidence and population-specific data

Real-world data further support the efficacy and safety of faricimab. Experts emphasize that real-world evidence strengthens the use of faricimab, as it consistently shows meaningful clinical benefits in diverse patient populations [27,42-44]. In the TRUCKEE study involving both treatment-naïve and previously treated patients with nAMD and DME, faricimab resulted in a significant mean CST reduction (−43.4 µm) and BCVA improvement after just three injections, with no cases of retinal vasculitis or arterial occlusion [38].

The CLEAR-DME study evaluated the real-world efficacy of faricimab in India, yielding promising results in the management of DME. The study echoed these findings, showing significant improvements in central macular thickness and BCVA at 6 months, with reductions in both intraretinal and SRF and no ocular or systemic adverse events [16]. This study is especially relevant for Indian patients, where patient adherence to frequent injections is a significant barrier. In India, the extended dosing intervals provided by faricimab could dramatically improve patient compliance and clinical outcomes. Experts recommend faricimab for its ability to reduce retinal fluid and improve vision early in treatment, particularly in populations with limited access to frequent injections [30,39,45].

In patients with PCV, the SALWEEN trial demonstrated that faricimab led to BCVA gains and 80% fluid resolution at Week 16, with no cases of retinal pigment epithelial tear or vascular occlusion [42]. A meta-analysis of seven studies covering 150 eyes with PCV confirmed these benefits, reporting a stable BCVA (−0.09 logMAR), central retinal thickness reduction (−169 µm), and 49% polyp closure with acceptable safety outcomes [46]. Experts strongly recommend faricimab for its efficacy in treating challenging conditions such as PCV, where conventional therapies may be less effective [6,46].

In the YOSEMITE and RHINE (DME) and TENAYA and LUCERNE (nAMD) trials, faricimab achieved non-inferior or superior visual and anatomical outcomes compared to aflibercept, while enabling extended dosing intervals of up to 16 weeks in the majority of patients. Importantly, its safety profile was comparable to that of existing anti-VEGF therapies, such as ranibizumab and aflibercept. These outcomes underpin its use in treatment-naïve and refractory cases and have been corroborated by emerging real-world data in Table 3.

**Table 3:** Studies on the efficacy and safety of faricimab in the management of patients with nAMD and DME BCVA: Best-corrected visual acuity; CMT: Central macular thickness; CST: Central subfield thickness; DME: Diabetic macular edema; IRF: Intraretinal fluid; nAMD: Neovascular age-related macular degeneration; PCV: Polypoidal choroidal vasculopathy; Q12W: Every 12 weeks; Q16W: Every 16 weeks; RCT: Randomized control trial; SRF: Subretinal fluid; T&E: Treat-and-extend regimen.

Trial name and design	Study population	Intervention	Outcomes	Safety outcomes
YOSEMITE/RHINE trials (Phase 3 RCTs) [26,29]	Patients with DME	Faricimab extended dosing intervals vs. aflibercept	Maintained BCVA and CST gains; >90% of patients achieved absence of DME (CST <325 μm).	Ocular adverse events were comparable with aflibercept.
Rhone-X (long-term extension trial of YOSEMITE/RHINE) [41]	Patients with DME from the YOSEMITE/RHINE trials	Faricimab extended dosing intervals	>90% of patients achieved absence of DME (CST<325 μm) with faricimab up to Q16W.	Well tolerated; the safety profile aligned with the YOSEMITE/RHINE trials.
TENAYA and LUCERNE trials (Phase 3 RCTs) [29]	Patients with nAMD	Faricimab (T&E) vs. aflibercept	Similar BCVA improvements at 2 years; a high proportion of patients (≥74%) achieved extended dosing intervals (Q12W/Q16W).	Ocular adverse events were comparable between faricimab and aflibercept.
SALWEEN trial (Phase 3b/4 study) [42]	Asians with PCV	Faricimab single-arm study with extended intervals	Improved BCVA; 80% of patients had no retinal fluid at week 16.	There were no cases of retinal pigment epithelial tear or retinal vascular occlusion events.
TRUCKEE study (retrospective interventional real-world study) [38]	Patients with nAMD and DME (both treatment-naïve)	Faricimab injections	After three injections, significant BCVA improvements were noted, along with CST reductions of −43.4 μm in all eyes.	There were two cases of intraocular inflammation and no cases of retinal vasculitis, retinal artery occlusion, or retinal pigment epithelium tears.
TAHOE study (multicenter, prospective study) [47]	Real-world patients with DME (both treatment-naïve and previously treated patients)	Faricimab injections	All eyes that received three injections of faricimab showed a notable improvement in BCVA and a decrease in CST of -45.28 μm.	No cases of intraocular inflammation, endophthalmitis, vasculitis, or retinal artery occlusion were observed.
CLEAR-DME study (multicenter observational study) [16]	Patients with DME in India (both treatment-naïve and previously treated patients)	Faricimab	Significant CMT reduction (<325 μm) and visual acuity gains (greater in the treatment-naïve group) at 6 months. Both IRF and SRF showed substantial reductions.	No ocular or systemic adverse events were noted.

Other real-world studies, such as TAHOE [47] and retrospective analyses [45,48], further affirm faricimab's value in recalcitrant and treatment-naïve eyes. These studies consistently show a reduction in CST and fluid, stabilization of vision, and absence of significant inflammatory or occlusive events.

Safety profile and comparative meta-analyses

Concerns around intraocular inflammation and vascular occlusion have historically limited the adoption of some anti-VEGF agents such as brolucizumab [32]. In this context, faricimab has demonstrated a favorable and consistent safety profile across pivotal clinical trials, meta-analyses, and real-world studies. Evidence from comparative analyses indicates that ocular adverse event rates, treatment discontinuation rates, and functional and anatomical outcomes with faricimab are comparable to those observed with established anti-VEGF agents [23,41,47,49,50]. Real-world observational data further support these findings, with low rates of intraocular inflammation and rare occurrences of retinal vasculitis reported in routine clinical practice [51,52]. These findings underscore faricimab’s safety in both clinical trials and routine practice. Quantitative safety outcomes are summarized in Table 4.

**Table 4:** Safety profile of faricimab: Quantitative evidence from clinical trials, meta-analyses, and real-world studies BCVA: Best-corrected visual acuity; CI: Confidence interval; CST: Central subfield thickness; EMR: Electronic medical record; IOI: Intraocular inflammation; T&E: Treat-and-extend; US: United States; VEGF: Vascular endothelial growth factor.

Evidence source	Safety outcome	Quantitative findings
Pivotal Phase 3 trials (TENAYA, LUCERNE, YOSEMITE, RHINE)	Overall ocular adverse events	Low incidence; comparable with aflibercept and ranibizumab
Network meta-analysis (26 studies)	Ocular adverse events	Comparable rates across faricimab T&E and other anti-VEGF regimens
	Treatment discontinuation	Low rates and comparable across treatment arms
Conventional meta-analysis	BCVA outcomes	No significant difference vs. other anti-VEGF agents
	CST outcomes	No significant difference vs. other anti-VEGF agents
	Adverse events	No significant difference vs. comparators
Moorfields Eye Hospital (real-world EMR data)	IOI per injection	0.19% (95% CI: 0.12-0.30)
	IOI per treated eye/patient	0.86% (95% CI: 0.53-1.33/0.49-1.39)
Vestrum Health Database (US)	Retinal vasculitis (± vascular occlusion)	0.008% of faricimab-treated eyes
Overall safety conclusion	—	Consistent and favorable safety profile across trials and real-world use

Clinical positioning of faricimab: expert panel recommendations

Based on the review of clinical trials and real-world data, the experts support faricimab as a first-line option for nAMD and DME. Its ability to target both VEGF-A and Ang-2 addresses key pathogenic mechanisms, providing durable anatomical and functional benefits. In addition, the experts highlighted patient adherence and access to healthcare as critical factors for successful implementation, particularly in India. Key points on the clinical utility and expert consensus regarding faricimab are summarized in Table 5.

**Table 5:** Evidence-based expert recommendations on faricimab as first-line treatment for nAMD and DME Ang-2: Angiopoietin-2; DME: Diabetic macular edema; nAMD: Neovascular age-related macular degeneration; PCV: Polypoidal choroidal vasculopathy; OCT: Optical coherence tomography; T&E: Treat-and-extend regimen; VEGF: Vascular endothelial growth factor.

Current anti-VEGF therapies effectively address vascular leakage but do not address other aspects of retinal diseases, including inflammation and fibrosis, which may contribute to vision loss [31-34].
Current anti-VEGF therapies inadequately address the Ang-2 signaling pathway in nAMD and DME. Faricimab is a bispecific molecule that targets two signaling pathways by inhibiting Ang-2 and VEGF-A, thereby improving vascular stability for durable efficacy. Dual inhibition of Ang-2 and VEGF with faricimab results in superior vascular stabilization, reduced leakage, inflammation, and decreased neovascularization [36,38,39].
Faricimab demonstrates greater retinal fluid reduction, improved clinical biomarkers, and extended treatment intervals compared to existing therapies [9,39,45].
Faricimab significantly improved visual acuity in Phase 3 clinical trials and real-world studies in DME and nAMD [27,42-44].
Early intervention with faricimab yields better outcomes, with a significant improvement in vision, faster resolution of fluid, and disease stabilization, requiring fewer injections and offering long-term durability. Faricimab enables disease stabilization even in recalcitrant cases resistant to conventional therapy [16,53].
In the SALWEEN study in treatment-naïve PCV patients, faricimab achieved complete regression of polypoidal lesions in 51% of patients and inactivation of polypoidal lesions in 86% of patients at just 16 weeks. This further establishes the significant role of faricimab in PCV, a subtype of nAMD, which is highly prevalent in the Indian population [42].
Clinical trials suggest that around 80% of patients maintained disease stability and vision gain with dosing intervals beyond 12 weeks, reducing treatment burden while preserving efficacy. Similarly, over 60% of patients achieved treatment intervals every 16 weeks in the Phase 3 clinical trials. OCT findings consistently showed anatomical improvements [26,29].
Personalized treatment using a T&E approach after "disease stability" is established with initial doses, aimed at individualizing the monitoring and treatment burden. Treatment before the worsening of nAMD and DME can potentially preserve visual acuity in the long term and reduce the frequency of clinic visits and injections [26].
Faricimab has established efficacy in Phase 3 clinical trials for treatment-naïve nAMD and treatment-naïve and previously treated DME patients (last treated ≥3 months before Day 1, limited to 25% of the total enrolment). Real-world studies have demonstrated the role of faricimab as a first-line treatment option for nAMD and DME. Long-term real-world studies from India will further establish the role of faricimab as a first-line treatment option [16,17,26,29,38,42,47].
Faricimab demonstrates improved clinical biomarkers in nAMD and DME [28,44].
Faricimab is well tolerated and has a favorable safety profile comparable to that of aflibercept and ranibizumab. Current data support its use for extended treatment intervals without compromising efficacy or safety [23,39,46,48].
In the Indian context, patient adherence and healthcare access limitations remain challenges for the widespread and early adoption of faricimab in treating nAMD and DME [2,16,40].

Way forward

Despite significant progress, retinal diseases remain a leading cause of vision loss worldwide, with increasing global prevalence [54-56]. Although anti-VEGF therapy is widely recognized as the standard of care for nAMD and DME, it has notable limitations [57]. Given that nAMD and DME are multifactorial diseases, optimizing patient outcomes may require a multifaceted treatment approach that goes beyond solely targeting VEGF [58]. Faricimab, by simultaneously inhibiting VEGF-A and Ang-2, offers a comprehensive approach, with clinical and real-world evidence supporting its efficacy, safety, and potential as a first-line treatment for both nAMD and DME.

Limitations

This manuscript presents expert opinion informed by published clinical trial data, meta-analyses, and real-world evidence, rather than a systematic review or formal guideline. Interpretations may therefore reflect the clinical experience of the participating experts and the evidence available at the time of discussion. Although pivotal trials and observational studies support the efficacy and safety of faricimab, long-term real-world outcomes, rare adverse events, and subgroup-specific responses may evolve with broader use. Variations in healthcare access, follow-up practices, and patient characteristics, particularly in resource-limited settings, may also influence outcomes. Accordingly, these recommendations are intended to support, not replace, individualized clinical decision-making.

## Conclusions

The Ang-2-Tie2 signaling pathway plays a crucial role in driving vascular instability in retinal diseases. By simultaneously targeting Ang-2 and VEGF-A, faricimab provides enhanced vascular stability, surpassing the effects of current anti-VEGF therapies and supporting its use as a first-line treatment for both nAMD and DME. Utilizing an individualized treatment approach with the T&E regimen could allow for better maintenance of vision gains and improved anatomical outcomes while reducing the frequency of injections. This strategy bridges the gap between the ideal clinical trial outcomes and real-world practice.

Faricimab represents a promising shift in the treatment paradigm for nAMD and DME, particularly in India, where challenges such as high diabetes prevalence, limited access to healthcare, and patient adherence challenges exist. Faricimab's dual pathway inhibition and extended dosing intervals make it an ideal first-line treatment for addressing these concerns. With the potential to reduce treatment burden and improve clinical outcomes and patient quality of life, faricimab may offer a patient-centric solution for managing retinal diseases in diverse populations.
